# Retraction: Improving healthcare workforce diversity

**DOI:** 10.3389/frhs.2024.1508057

**Published:** 2024-10-16

**Authors:** Frontiers Editorial Office

**A Retraction of the Opinion**
Improving healthcare workforce diversity By Zou Y (2023). Front. Health Serv. 3:1082261. doi: 10.3389/frhs.2023.1082261

Following publication, concerns were raised regarding the scientific validity of the article. An investigation was conducted in accordance with Frontiers’ policies.

It was found that the concerns were valid and that the article does not meet the standards of editorial and scientific soundness for Frontiers in Health Services; therefore, the article has been retracted.

The retraction was approved by the Chief Editors for Frontiers in Health Services and the Chief Executive Editor of Frontiers. The author does approve of the retraction.

